# Chytridiomycosis Outbreak in a Chilean Giant Frog (*Calyptocephalella gayi*) Captive Breeding Program: Genomic Characterization and Pathological Findings

**DOI:** 10.3389/fvets.2021.733357

**Published:** 2021-09-24

**Authors:** Mario Alvarado-Rybak, Paz Acuña, Alexandra Peñafiel-Ricaurte, Thomas R. Sewell, Simon J. O'Hanlon, Matthew C. Fisher, Andres Valenzuela-Sánchez, Andrew A. Cunningham, Claudio Azat

**Affiliations:** ^1^Sustainability Research Centre & PhD Program in Conservation Medicine, Faculty of Life Sciences, Universidad Andres Bello, Santiago, Chile; ^2^Institute of Zoology, Zoological Society of London, London, United Kingdom; ^3^Núcleo de Ciencias Aplicadas en Ciencias Veterinarias y Agronómicas, Universidad de las Américas, Santiago, Chile; ^4^Criadero y Centro de Exhibición de la Rana Chilena Calyptocephalella gayi, Santiago, Chile; ^5^Department of Infectious Disease Epidemiology, MRC Centre for Global Infectious Disease Analysis, School of Public Health, Imperial College London, London, United Kingdom; ^6^ONG Ranita de Darwin, Valdivia, Chile; ^7^Instituto de Conservación, Biodiversidad y Territorio, Facultad de Ciencias Forestales y Recursos Naturales, Universidad Austral de Chile, Valdivia, Chile

**Keywords:** agnathia, amphibians, *Batrachochytrium dendrobatidis*, BdGPL, brachygnathia, Chile, emerging infectious disease, whole-genome sequencing

## Abstract

Emerging infectious diseases in wildlife are increasingly associated with animal mortality and species declines, but their source and genetic characterization often remains elusive. Amphibian chytridiomycosis, caused by the fungus *Batrachochytrium dendrobatidis* (*Bd*), has been associated with catastrophic and well-documented amphibian population declines and extinctions at the global scale. We used histology and whole-genome sequencing to describe the lesions caused by, and the genetic variability of, two *Bd* isolates obtained from a mass mortality event in a captive population of the threatened Chilean giant frog (*Calyptocephalella gayi*). This was the first time an association between *Bd* and high mortality had been detected in this charismatic and declining frog species. Pathological examinations revealed that 30 dead metamorphosed frogs presented agnathia or brachygnathia, a condition that is reported for the first time in association with chytridiomycosis. Phylogenomic analyses revealed that *Bd* isolates (PA1 and PA2) from captive *C. gayi* group with other *Bd* isolates (AVS2, AVS4, and AVS7) forming a single highly supported Chilean *Bd* clade within the global panzootic lineage of *Bd* (*Bd*GPL). These findings are important to inform the strengthening of biosecurity measures to prevent the impacts of chytridiomycosis in captive breeding programs elsewhere.

## Introduction

Although amphibian enigmatic declines had been identified by herpetologists as early as the 1970s, they were only recognized two decades later as a global phenomenon that in some cases could not be explained by environmental changes or other expected anthropogenic factors alone (1–3). The discovery of the amphibian-killing fungus *Batrachochytrium dendrobatidis* [hereafter *Bd* (4, 5)] was a turning point in understanding why many amphibian species have been in steep decline. The emergence of *Bd*, which causes the lethal disease, amphibian chytridiomycosis, has been associated with amphibian population declines of more than 500 species, including the presumed extinction of at least 90 species (6). Evidence suggests that *Bd* recently spread across the globe from an endemic focus, with East Asia as the most likely source from where it expanded to other continents during the past century (7–10). Its global spread has been mainly facilitated by the international trade of amphibians, particularly the North American bullfrog (*Lithobates catesbeianus*), the most intensively farmed frog worldwide (11–15).

Novel genomic techniques, including whole-genome sequencing and multilocus sequence typing have shown the existence of at least five major lineages of *Bd*: *Bd*GPL, *Bd*CAPE, *Bd*ASIA-1 (including *Bd*CH), *Bd*ASIA-2/*Bd*BRAZIL and *Bd*ASIA-3 (9, 10, 16). Of these, the global panzootic lineage (*Bd*GPL) is the most widespread variant of *Bd* and is responsible for most known cases of amphibian population declines due to chytridiomycosis (9). Although *Bd*GPL is highly virulent, its impacts are context-dependent (17), and under some conditions other lineages may be responsible for lethal disease and population declines (18). Additionally, multiple introductions of *Bd* have led to different lineages of *Bd* coming into contact, resulting in the formation of interlineage recombinants (e.g., through the co-infection of amphibians) that may have higher pathogenicity or transmissibility (9, 10, 14, 16, 19, 20). For instance, interlineage recombinants have been reported for *Bd*GPL with *Bd*ASIA-2/*Bd*BRAZIL, and for *Bd*GPL with *Bd*CAPE (9, 10, 14, 19).

Despite South America being the region with the greatest loss of biodiversity due to *Bd* (6), only low numbers of *Bd* isolates from this region have been genetically characterized (14, 21–26). This limits our capacity to adequately understand the epidemiological processes that have led to impacts of chytridiomycosis on South American native amphibians. Our focus here, the Chilean giant frog (*Calyptocephalella gayi*), is endemic to Chile and is considered as a living fossil since its family represents an old neobatrachian clade that diverged during the Cretaceous around 100–120 Mya (27). With females reaching up to 2 kg, this is the second largest anuran species worldwide, which has led this species to be of economic interest as a food source (28). This highly aquatic species is considered Vulnerable by the IUCN Red List, and is threatened by overconsumption, habitat loss due to agriculture, and invasive species including several introduced fish and the African clawed frog [*Xenopus laevis* (29)]. Chytridiomycosis has been suspected to be a contributing factor in its steep decline (30), but to date, no evidence has been found linking *Bd* to lethal effects in *C. gayi*. Based on histology and whole-genome sequencing, the aim of this study is to describe the lesions of chytridiomycosis in *C. gayi* and the genetic characterization of *Bd* isolates obtained from a chytridiomycosis outbreak that occurred in a *C. gayi* captive breeding program in Chile. In addition, the genomics of *Bd* isolates from captive *C. gayi* together with previously obtained Chilean *Bd* isolates are compared with a global panel of *Bd*.

## Materials and Methods

### Chilean Giant Frog Captive Breeding Center

The Chilean giant frog captive breeding center (Resolution N°2358/2013 by the Chilean Agriculture and Livestock Service) in Santiago has been functioning since 2013, with its objective to generate reproductive knowledge and to support the conservation of *C. gayi*. The center was built in an area of 70 m^2^ and was composed of 10 large and 10 small tanks for tadpoles (100 and 30 L each, respectively), 10 small tanks for recently metamorphosed frogs (30 L) and 20 medium size tanks for adult frogs (50 L). Water used in tanks was from the mains supply but had been left to stand for 2 days to allow chlorine evaporation. Around 50% of water in the tanks was changed twice a week. Tadpoles were fed on spirulina algae supplemented with lettuce given *ad libitum*, while postmetamorphs and adults were fed twice a day with dried amphipod crustaceans (*Orchistoidea* spp.) supplemented with chicken protein, vitamins, and minerals. By August 2016, the captive breeding program comprised ~400 1-year-old tadpoles, six juveniles, and 86 breeding adults (43 females and 43 males).

### Mortality and Pathological Analyses

In September 2016, the program received new individuals from a separate *C. gayi* captive breeding program that had been terminated. The newly incoming individuals consisted of 800 2-year-old tadpoles and 18 breeding adults (9 females, 9 males), which were maintained in separate tanks from the resident animals. By early November 2016, 40 of the new tadpoles completed metamorphosis. From December 2016 to January 2017, 75 of the new individuals died: 37 tadpoles, 35 postmetamorphs and three reproductive adults. All dead postmetamorphs had not consumed any food after metamorphosis, and some of the tadpoles were observed to be lethargic prior to death and exhibiting partial depigmentation of the mouthparts, a finding consistent with amphibian chytridiomycosis (31). Freshly dead animals (4 tadpoles and 29 postmetamorphs) were transported in refrigerated conditions to the laboratory for postmortem examination and *Bd* isolation. Necropsies of the 29 postmetamorphs were performed according to standard protocol (32). Tissue sections of postmetamorphs were collected in neutral-buffered 10% formaldehyde from any organ displaying gross lesions and from lung, liver, spleen, kidney, skeletal muscle, heart, skin, stomach, small and large intestines. For histopathological analyses, tissues were embedded in paraffin wax, sectioned (4–5 μm) and stained with hematoxylin and eosin (H&E).

### *Bd* Sampling and qPCR Assay

Non-invasive skin swabs (MW100, Medical & Wire Equipment Co.) were obtained from postmetamorphic amphibians (*n* = 29) by firmly running the swabs five times each over the ventral abdomen and pelvis, each ventral hind limb (femur and tibia) and the plantar surface of each hind foot (33). Also, from dead tadpoles (*n* = 4), samples were obtained from the oral disc by rotating the swab 10 times around the mouth opening. Swabs were kept in a cool box until freezing at −80°C once back at the laboratory. Briefly, DNA extraction from skin and oral swabs and subsequent detection of *Bd* DNA using a specific real time qPCR assay was done following Soto-Azat et al. (34). For each sample, diagnostic assays were performed in duplicate, and standards of known zoospore concentration (obtained from a previous *Bd* culture) were included within each PCR plate as positive controls. We assumed that a *Bd*-positive swab was indicative of *Bd* infection. By including known concentrations of *Bd* DNA in serial diluted positive control wells on each PCR plate, we were able to quantify infection intensity, which we defined as the number of zoospore equivalents/swab (ZE). To quantify and correct the infection intensity per swab, each genomic value obtained from the qPCR assay was multiplied by 120 to account for sample dilution (35).

### *Bd* Isolation

Freshly dead tadpoles with suspected *Bd* infection (*n* = 4) were used for *Bd* isolation following Longcore et al. (5) and Fisher et al. (36). Subsequent confirmation of *Bd* infection status and load by qPCR, served to guide *Bd* isolation efforts. Within 8 h. after death, the whole mouthparts of *Bd*-positive dead tadpoles were removed and sectioned into small pieces and deposited in a fungal growth TGhL medium (8 g. tryptone, 2 g. gelatin hydrolysate, 4 g. lactose, 10 g. agar). Cultured sections were first cleaned using an agar plate with antibiotics (200 mg/L penicillin-G and 400 mg/L streptomycin sulfate), and then placed singly into TGhL agar plate with antibiotics incubated at 15–20°C. Because zoospore release may occur immediately, especially from tadpole mouthparts, cultures were examined with an inverted microscope for the presence of active zoospores every day for up to 1 week. Once growth of zoospores and/or zoosporangia was observed, part of the agar was transferred to a new TGhL agar plate without antibiotics and incubated at 15–20°C up to 1 week. Isolates were then passaged no more than three times in order to lessen the chance of genomic change due to prolonged laboratory culture (37).

### DNA Extraction, Sequence Library Preparation and Phylogenomic Analyses

We performed DNA extraction using the MasterPure^TM^ Yeast DNA Purification Kit (Epicentre, Wisconsin, USA) from all obtained purified *Bd* cultures. DNA extractions were first quantified using a Tapestation 2200 (Agilent Technologies, California, USA) and Qubit 2.0 fluorimeter (Thermo Fisher Scientific, Massachusetts, USA), and then sequenced using an Illumina HiSeq 2000 (Illumina, California, USA). Subsequently, TruSeq Nano 350 gel-free sequencing libraries were prepared for 125 + 125 bp paired-end sequencing using Illumina HiSeq high output v4 chemistry, and sequencing reads cleaned of adapter sequences and quality trimmed using cutadapt v1.10 (38). Reads of the JEL423 reference genome (GenBank assembly accession: GCA_000149865.1) were mapped using Burrows-Wheeler Aligner v0.7.8 (39).

We processed the resulting sequence alignment map (SAM) files using SAMtools v1.3.1 with the “fixmate” and “sort” programs to read the files for variants discovery. We performed the variant detection in a two-step process using Freebayes version dbb6160 (40). First, sorted SAM files for each isolate in the phylogeny were independently called to find variant positions and merged into a single variant call format (VCF) file. Second, each of the samples was re-called using the positions in VCF file, to produce a squared-off call set (a genotype call was made at every locus for each isolate, including for missing data). All VCF files were processed using vcflib (41) to break complex variants into allelic primitives and vt (42) to normalize short insertion and deletion sequences. VCF files were then quality filtered using bcftools v1.3.1 (43) to accept only variants with sufficient supporting evidence. Potentially polymorphic sites were filtered using the settings in the bcbio.variation.recall squaring-off pipeline (44), with sites not passing these filters set to homozygous reference (there was not enough evidence to call a variant at that position). Then, the processed VCF files were merged into a single multisample VCF and extracted a FASTA file of the SNP variant calls. Phylogenomic analyses were conducted using RAxML v8.2.9 with GTRCAT model with 500 bootstrap runs. Weir and Cockerham's estimator was performed using a sliding-window comparison of F_ST_ of Chilean *Bd* isolates against a global panel of representative global diversity of *Bd* in vcftools. Single nucleotide polymorphisms (SNPs) that were in high linkage disequilibrium were pruned from the dataset using the SNPRelate package version 1.10.2 in R v3.4.0 (45). After pruning using a sliding-window based analysis and a linkage disequilibrium threshold of 0.125, 3,900 SNPs positions remained which were analyzed using SNPRelate and plotted with ggplot2 (46). Finally, the clustering of Chilean *Bd* isolates against a global panel of *Bd* was investigated using principal component analysis (PCA) with adegenet package (47) and plotted with ggplot2 (46).

## Results

### Pathology and *Bd* qPCR Assay

**Figure 1:**
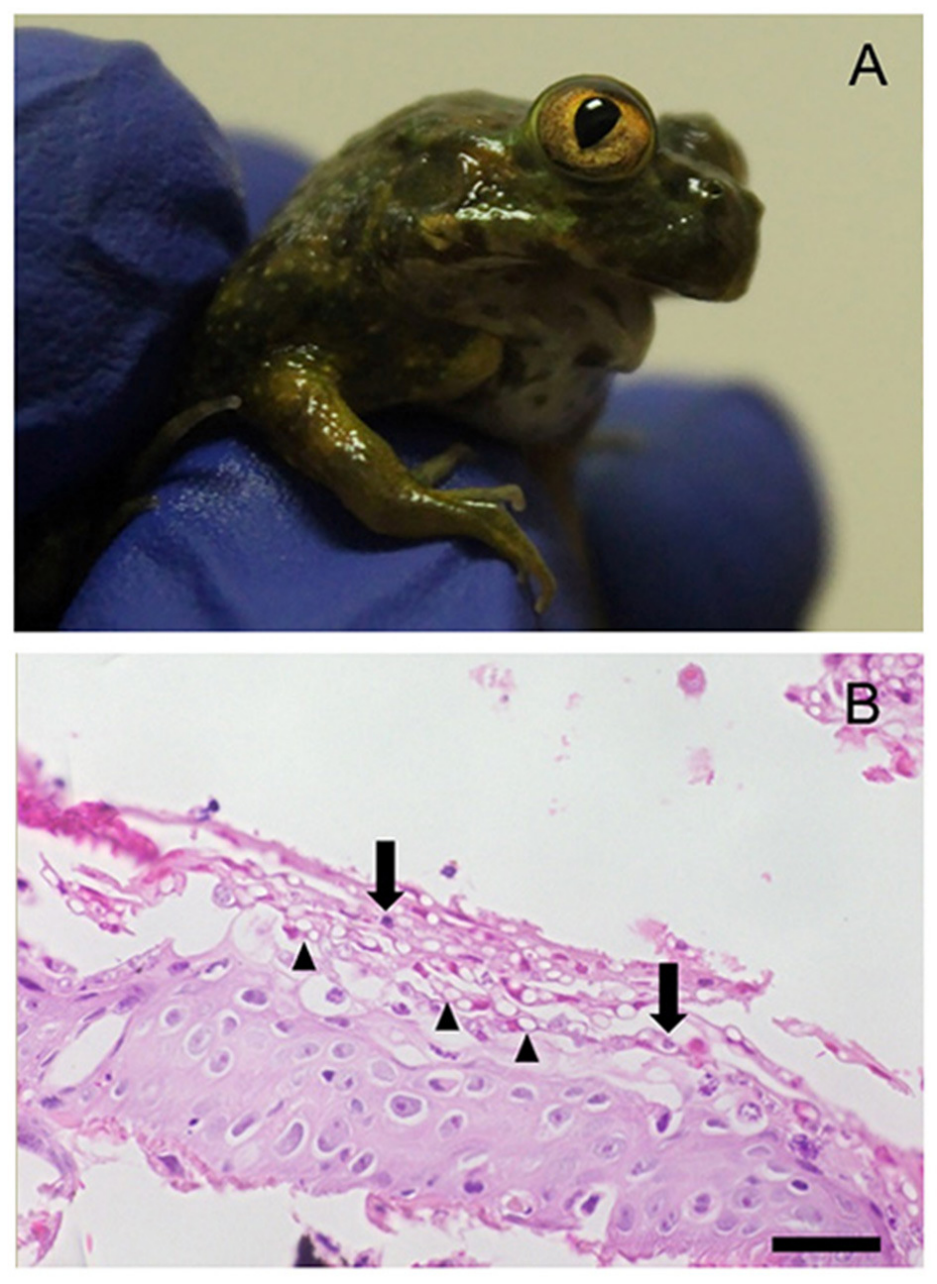
A Chilean giant frog (*Calyptocephalella gayi*) postmetamorph showing signs of disease. **(A)** Absence of jaw (agnathia). **(B)** Histological section of hind limb skin. Note distinct stages of developing zoosporangia (arrows) and multiple empty spaces (arrowheads) within the superficial keratinized layer, morphologically typical of *Batrachochytrium dendrobatidis* infection. Stained with hematoxylin and eosin. Bar = 24 μm (bottom).

During December 2016 and January 2017, we observed a mass mortality event in a *C. gayi* captive breeding program, killing 87.5% (35/40) of metamorphosed frogs in the newly acquired group of animals. Of these, 30 individuals presented: jaw deformation (*n* = 21) or absence (*n* = 9) of oral structures, dying a few weeks after completing metamorphosis as they could not feed properly (Figure 1A). PCR *Bd*-positive samples were detected in 100% of sampled tadpoles, and postmetamorphic individuals (*n* = 33). The infection load in *Bd*-positive amphibians ranged from 95 to 147,366 ZE (median = 9,842). Of the total number of infected frogs, 33.3% (11/33) had more than 10,000 ZE. At gross necropsy a distended gallbladder (with bile) and the absence of gastrointestinal content was observed in all individuals with jaw absence/deformation (30/35). We confirmed *Bd* infection microscopically, as we observed hyperkeratosis within the superficial layer of the skin with distinct stages of developing zoosporangia that are morphologically typical of *Bd* (Figure 1B). No other macroscopic or microscopic findings were observed in the other analyzed tissues.

### *Bd* Isolation and Phylogenomic Analyses

**Figure 2:**
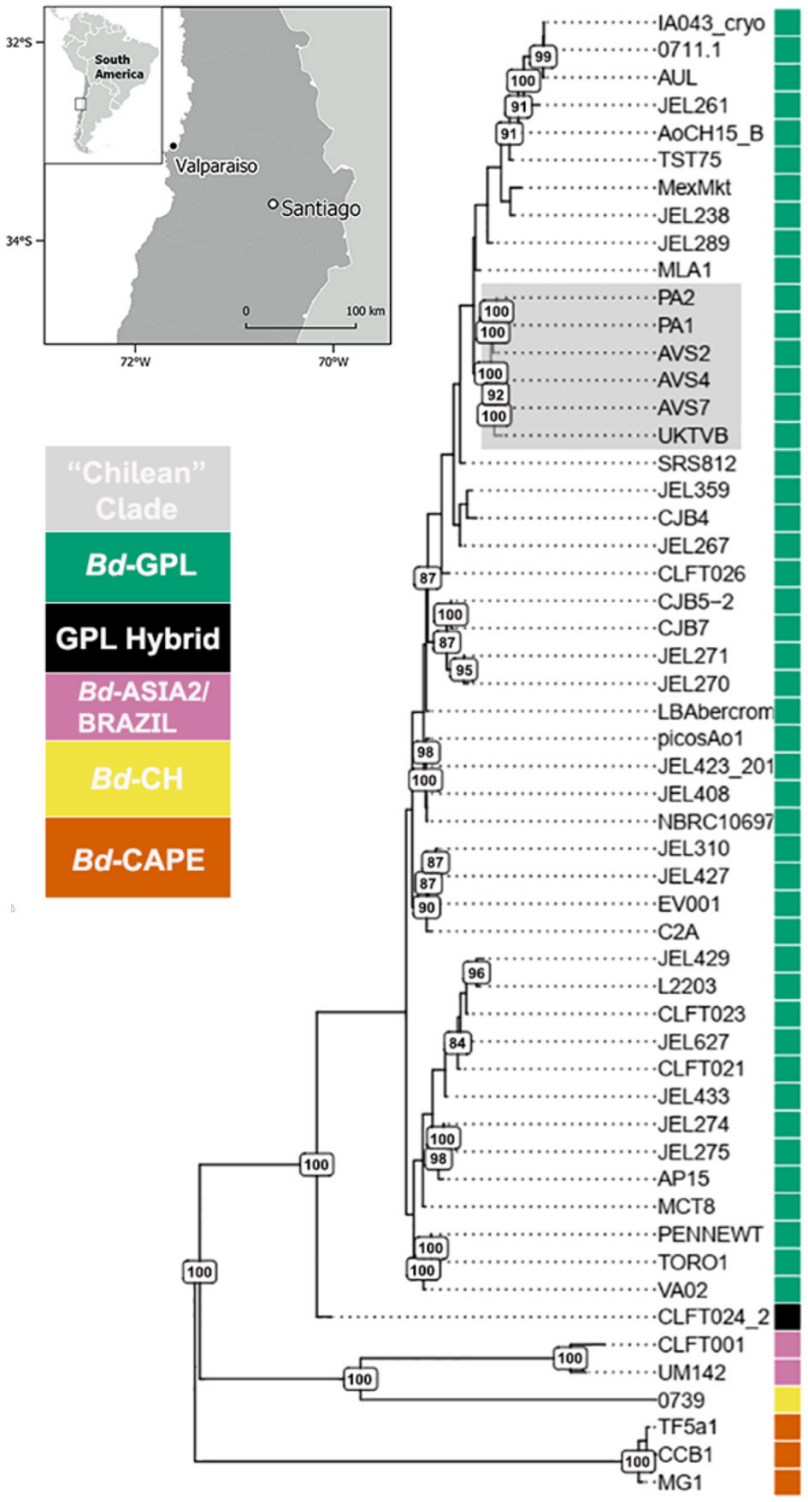
Global phylogeny of 54 isolates of *Batrachochytrium dendrobatidis* (Bd) based on 363,497 segregating sites obtained by whole genome sequencing. The clade containing the Bd isolates PA1 and PA2 from captive *C. gayi* grouping with other Bd isolates from Chile and the United Kingdom is highlighted in gray. The branches of the tree are weighted (thickness) by bootstrap support (500 replicates), with branches with 80% of support and above labeled.

From our attempts to culture *Bd* from four freshly dead *C. gayi* tadpoles, we obtained two isolates (PA1 and PA2, WGS read data available at the NCBI Sequence Read Archive: https://www.ncbi.nlm.nih.gov/sra under accession numbers SRS8215364 and SRS8215364 and SRS8216816, respectively). Phylogenomic analyses showed that our *C. gayi* isolates grouped within *Bd*GPL forming a single and highly supported clade (100% bootstrap support; Figure 2). The *Bd* isolates were fixed for 99.4% of the segregating sites that were observed in *Bd*GPL after filtering for missing positions. There were only 2,257 variable sites exclusive to the *C. gayi Bd* isolates. Although we compared the genomes of *C. gayi* isolates (PA1 and PA2) with an extensive global panel of *Bd*, they were shown to be highly divergent from the only known regional potentially endemic lineage in South America (*Bd*Asia2/*Bd*Brazil). Within *Bd*GPL, the *Bd* isolates from captive *C. gayi* clustered with other isolates obtained from wild amphibians in Chile (AVS2 from *Batrachyla antartandica*, AVS4 from *Xenopus laevis* and AVS7 from *C. gayi*) and an isolate from the UK (UKTvB), collected from a smooth newt (*Lissotriton vulgaris*) in 2009 in Kent, United Kingdom, to form a well-supported clade (100% bootstrap support; Figure 2).

**Figure 3:**
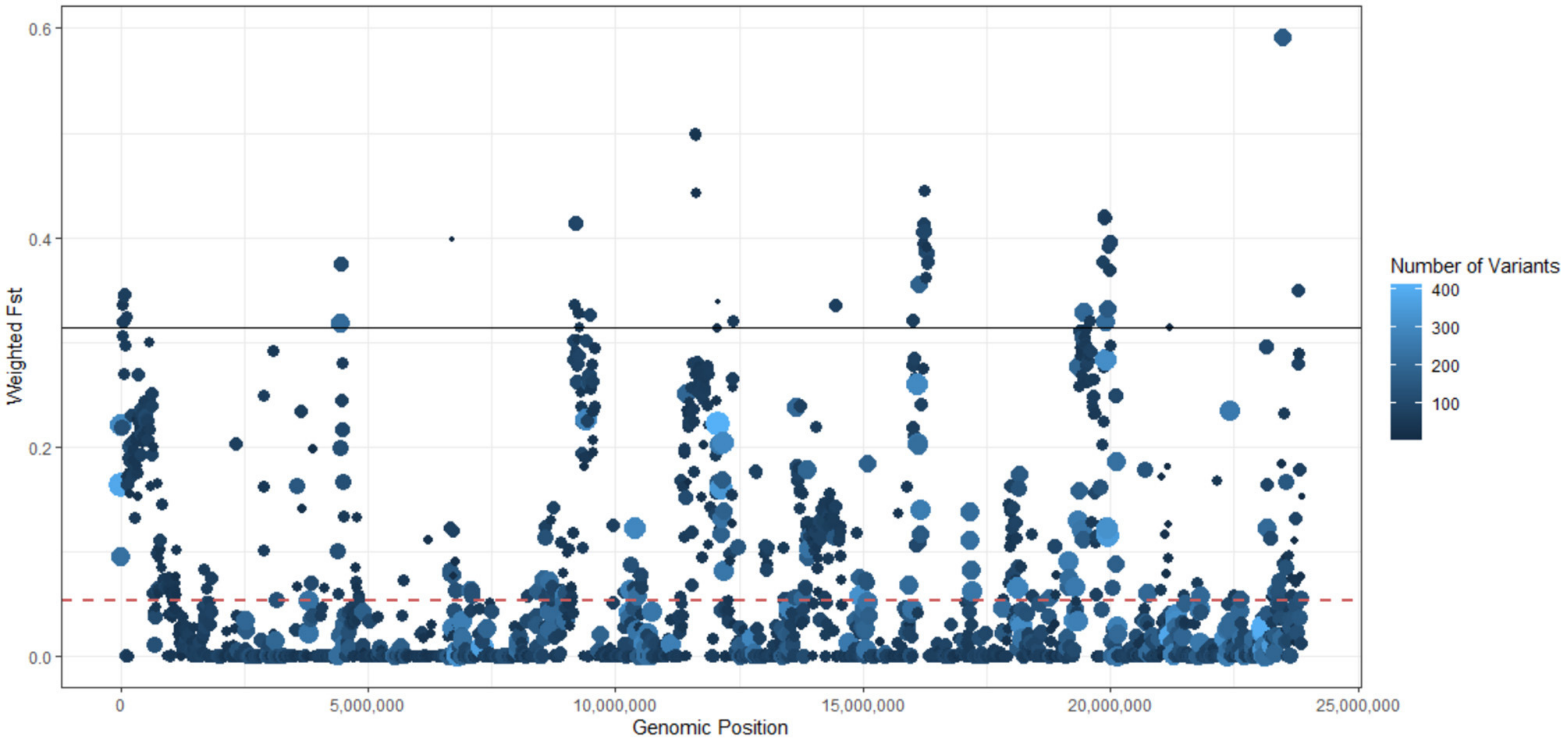
Sliding window analysis of population differentiation of *Batrachochytrium dendrobatidis* (*Bd*) isolates (PA1 and PA2) against other 45 global panzootic lineage of *Bd* (*Bd*GPL) isolates using Weir and Cockerham's F_ST_ estimator. Each point represents a 10 Kb genomic window, with a 5 kb step-size. The dashed red line represents the mean F_ST_ (0.0538). The solid black line represents the 95% quantile threshold of the F_ST_ estimator (0.3141). Each point is sized and colored on a log scale by the number of variants in each window. The legend indicates the color scale (the number of SNPs included in each window varied from 1 to 364, with a median of 31). Point size from small to large is scaled from low to high numbers of variants.

**Figure 4:**
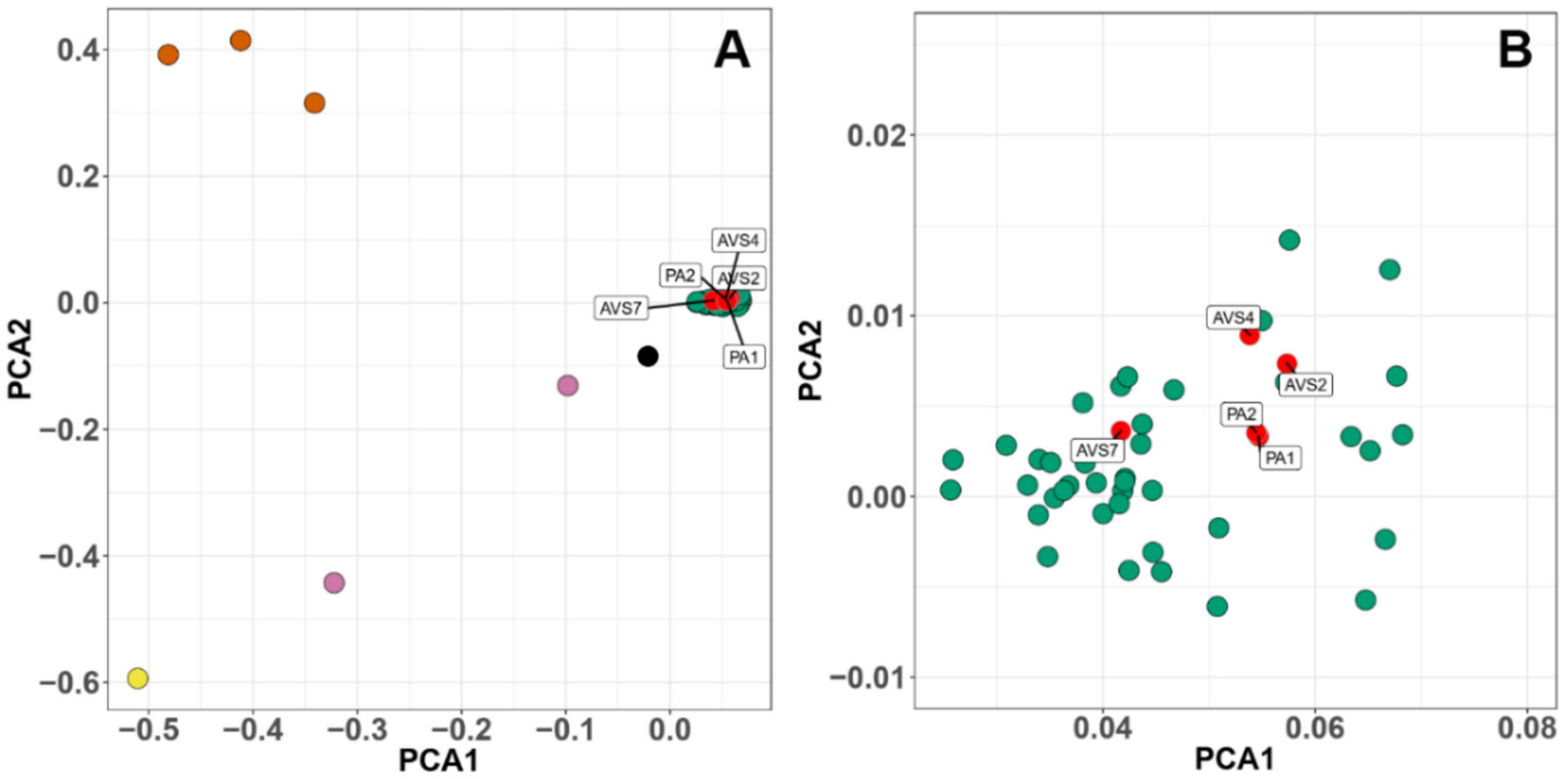
Principal components analysis (PCA) of 3,900 SNPs in linkage equilibrium of a global panel of 54 *Batrachochytrium dendrobatidis* (Bd) isolates. Each point represents an isolate, colored by phylogenetic lineage. **(A)** Bd isolates separate into clearly defined clusters. **(B)** Higher resolution of the global panzootic lineage of Bd (BdGPL) cluster showing the position of the Chilean isolates within the cluster. The axes plot the first and second principal components, PCA1 and PCA2. Chilean isolates are clustered within BdGPL. Chilean isolates (red), other BdGPL (green), BdASIA2/BdBRAZIL (pink), hybrids between the previous two groups (black), BdCH (yellow), BdCAPE (orange).

We used the Weir and Cockerham's estimator to perform a sliding-window comparison of F_ST_ of *Bd* isolates (PA1 and PA1) against all the other *Bd*GPL isolates. In this analysis, we identified several stretches of genome where the F_ST_ estimator was more than two standard deviations greater than the mean of all F_ST_ values, indicating differentiation due to positive selection or reduced rates of recombination (Figure 3). Finally, we analyzed isolate clustering using PCA on a filtered subset of 3,900 SNPs in linkage equilibrium, revealing an overall population structure that is consistent with our phylogenetic analyses (Figure 4).

## Discussion

The now globalized *Bd* has caused the greatest loss of biodiversity known due to a single pathogen (6). The impacts of chytridiomycosis have likely been underestimated, as the affected amphibian species are often difficult to study, particularly in endangered cryptic species that occur in remote locations (48). In addition, not all *Bd* lineages have the same impact on infected amphibian populations and species, therefore a better understanding of the genetic diversity of *Bd* is critical to understanding the risk presented by this pathogen and to informing mitigation actions (16). In this study, we describe a mass mortality event due to chytridiomycosis in an endangered species of amphibian in a captive breeding program. We genetically characterized two *Bd* isolates from this outbreak, showing that they nested within the *Bd*GPL clade and were highly related to *Bd* genotypes previously isolated from wild amphibians in Chile.

The presence of agnathia and brachygnathia associated with *Bd* infection in postmetamorphic amphibians has not been reported before. Tadpole oral malformations have been associated with low temperatures (49), water contamination (50), nutrition (51), or ecological factors (52, 53). Although, this malformation might have been due to an unknown environmental or other cause, it is likely associated with *Bd* infection of the oral discs of tadpoles (54–56). Absence or reduction in development of the lower jaw may have had a profound impact on the ability of postmetamorphic amphibians (and tadpoles) to acquire food, contributing to death along with chytridiomycosis. The presence of distended gall bladders and the absence of gastrointestinal content in all the animals examined suggests a lack of feeding. Although oral deformations in postmetamorphic amphibians have not been used before as an indication of chytridiomycosis, they might be still important and may indicate an unknown sequela of *Bd i*nfection that may have been overlooked previously. This study highlights the need to use accurate diagnostic techniques such as qPCR or histology to be able to complement these observations (57).

Captive breeding has increasingly been used as a tool for amphibian conservation, but for these initiatives to be successful, several aspects must be considered, including genetic management and biosecurity protocols (58, 59). In our case, newly admitted individuals of *C. gayi* came from a semi-open captive breeding program that was supplied with water from an agricultural canal, in which *X. laevis* had previously been recorded (34). Despite the implementation of quarantine, this was not enough to prevent the introduction of *Bd* to the captive breeding program, causing mortality in the newly admitted animals. This highlights the importance of implementing strict biosecurity protocols against *Bd* (and other pathogens), such as *Bd* testing prior to admittance, preventive antifungal treatment or disinfection of water and materials (59).

Susceptibility to *Bd* is also influenced by environmental factors, such as climate (20). Immune function in amphibians is closely dependent on environmental temperature (60). For instance, low temperature has been associated with a lower survival in *Bd*-exposed frogs under laboratory conditions (60, 61) and chytridiomycosis die-offs have been often associated with higher elevation, lower temperature and winter season (62–64). It is possible that in our case, stress associated with transportation might have induced immunosuppression, facilitating the development of chytridiomycosis. Ramsey et al. (65) demonstrated that natural resistance to *Bd* in *X. laevis* can be reversed with the implementation of sub-lethal immunosuppressive treatments, such as exposure to X-irradiation or norepinephrine injections.

The *Bd* lineage that we isolated from infected individuals in this chytridiomycosis outbreak, *Bd*GPL, has been associated with catastrophic mass mortalities and population declines in multiple continents (22, 48, 66, 67). Over the last four decades, a severe population decline of two species of Darwin's frogs (*Rhinoderma darwinii* and *R. rufum*) populations has occurred across central and south Chile. Retrospective and cross-sectional *Bd* studies and population monitoring data suggest that chytridiomycosis has contributed to these extirpations (33, 48) and that *Bd* has been present in Chile at least since 1970 (33). Although characterization of *Bd* isolates infecting *R. darwinii* has not been achieved so far, the identification of *Bd*GPL over a large area of Chile, provides support to the hypothesis that BdGPL is causing these declines (25).

In our phylogenomic whole-genome analysis, all the Chilean *Bd* isolates (including the two isolates from captive *C. gayi*) grouped together with a genotype isolated in 2009 from the United Kingdom (UKTvB). A similar phylogenetic relationship was observed when restricting the analysis to a subset of the genome spanning a heterozygosity loss event shared by all *Bd*GPL isolates, but in this case, isolates from other European countries and a Canadian isolate also group with the Chilean isolates (25). While there has been no report of mortality in the wild caused by UKTvB, a challenge with this isolate in the Mallorcan midwife toad (*Alytes muletensis*) under laboratory conditions caused a 73% mortality rate (18). It is likely, therefore, that the *Bd*GPL genotypes in Chile are virulent and have caused amphibian mortalities in nature (25). However, detecting mortalities in the wild is often difficult, particularly in cryptic species, for which better surveillance is needed. In addition, we also evaluated the presence of *Frog virus 3* (FV3) ranavirus in the same individuals as a possible cause of mortality, however all tested negative using a qPCR assay. Previously, a mass mortality of adult *C. gayi*, allegedly due to a drought, was described in central Chile, however whether *Bd* was involved in this mortality event is unknown as no fresh carcasses were available for necropsy, *Bd* detection or other diagnostics to be performed (68).

The low number of segregating sites exclusive to the Chilean *Bd* isolates, compared to the total number of sites where the *Bd*GPL isolates are polymorphic, suggests a single and recent introduction of *Bd* into Chile, possibly through the international movement of amphibians, other aquatic animals, fomites, or tourism (9, 14, 25, 66). Molecular characterization of further isolates from non-surveyed areas in Chile and neighboring countries (e.g., Argentina and Peru), along with the calibration of a genome-wide molecular clock, is required to confirm this hypothesis. The existence of an apparently unique and recently introduced lineage of *Bd* in Chile differs with the known history of this pathogen in Brazil, where both *Bd*GPL and *Bd*Asia2/*Bd*Brazil coexist, with evidence of multiple interlineage recombination events between them (9, 24). Therefore, together with the potential introduction of novel *Bd* genotypes, interlineage recombination can potentially arise facilitated by the globalization of human and animal transport (16). This highlights the importance of biosecurity measures at the national and local level, to prevent the introduction and establishment of further pathogenic *Bd* lineages, as this pathogen has the capability to increase its genomic diversity through the exchange of haplotypes among lineages (25).

Our work describes for the first time a mass mortality event in the endangered giant Chilean frog from a captive breeding program. We also provide new data on the potential susceptibility of *C. gayi* to the impacts of chytridiomycosis, a species that has been declining fast across its distribution in Chile. The high mortality observed in *C. gayi* with postmetamorphs exhibiting agnathia or brachygnathia as a possible consequence of oral infection with *Bd* in tadpoles has not been described previously and is a condition that can be considered in the monitoring of amphibians maintained in captivity, such as farms, zoos and *ex situ* conservation programs. We described two new isolates of *Bd* in Chile, belonging to *Bd*GPL and clustering in a single group with another three previously isolated *Bd* isolates from central and south Chile (9, 25), for which evidence as a cause of amphibian mortality and population declines is growing.

## Data Availability Statement

The datasets presented in this study can be found in online repositories. The names of the repository/repositories and accession number(s) can be found at: NCBI SRA; SRS8215364 and SRS8216816.

## Ethics Statement

The animal study was reviewed and approved by Bioethics Committee Universidad Andres Bello and by the Zoological Society of London's Ethics Committee.

## Author Contributions

MA-R and CA led the manuscript writing. MA-R, PA, and AP-R collected the data. MA-R and AP-R completed the PCR analysis, post-mortem investigation, and Bd isolation. TS, SO'H, and MF competed the whole genome analyses. AV-S, AC, and CA supported data analyses and pathological research. All authors contributed to the manuscript.

## Funding

This study was funded by FONDECYT Regular number 1211587 (to CA), and the Dirección General de Investigación y Doctorados, Universidad Andres Bello, grant N ° DI-582-14/I (to MA-R). TS and MF were supported by a grant from Natural Environmental Research Council (NERC; NE/S000844/1) and the UK Medical Research Council (MRC; MR/R015600/1). MF is a fellow in the CIFAR Fungal Kingdoms program.

## Conflict of Interest

The authors declare that the research was conducted in the absence of any commercial or financial relationships that could be construed as a potential conflict of interest.

## Publisher's Note

All claims expressed in this article are solely those of the authors and do not necessarily represent those of their affiliated organizations, or those of the publisher, the editors and the reviewers. Any product that may be evaluated in this article, or claim that may be made by its manufacturer, is not guaranteed or endorsed by the publisher.
